# Correlating data from different sensors to increase the positive predictive value of alarms: an empiric assessment

**DOI:** 10.12688/f1000research.1-45.v1

**Published:** 2012-11-08

**Authors:** Yuval Bitan, Michael F O’Connor

**Affiliations:** 1 Cognitive Technologies Laboratory, The University of Chicago, Chicago, IL, USA; 2 Department of Anesthesia and Critical Care, The University of Chicago, Chicago, IL, USA

## Abstract

**Objectives:** Alarm fatigue from high false alarm rate is a well described phenomenon in the intensive care unit (ICU). Progress to further reduce false alarms must employ a new strategy. Highly sensitive alarms invariably have a very high false alarm rate. Clinically useful alarms have a high Positive-Predictive Value. Our goal is to demonstrate one approach to suppressing false alarms using an algorithm that correlates information across sensors and replicates the ways that human evaluators discriminate artifact from real signal.

**Methods:** After obtaining IRB approval and waiver of informed consent, a set of definitions, (hypovolemia, left ventricular shock, tamponade, hemodynamically significant ventricular tachycardia, and hemodynamically significant supraventricular tachycardia), were installed in the monitors in a 10 bed cardiothoracic ICU and evaluated over an 85 day study period. The logic of the algorithms was intended to replicate the logic of practitioners, and correlated information across sensors in a way similar to that used by practitioners. The performance of the alarms was evaluated via a daily interview with the ICU attending and review of the tracings recorded over the previous 24 hours in the monitor. True alarms and false alarms were identified by an expert clinician, and the performance of the algorithms evaluated using the standard definitions of sensitivity, specificity, positive predictive value, and negative predictive value.

**Results:** Between 1 and 221 instances of defined events occurred over the duration of the study, and the positive predictive value of the definitions varied between 4.1% and 84%.

**Conclusions:** Correlation of information across alarms can suppress artifact, increase the positive predictive value of alarms, and can employ more sophisticated definitions of alarm events than present single-sensor based systems.

## Introduction

Historically, desire for high performance and concern over legal liability has motivated the design of alarm systems in clinical medicine that are highly sensitive, but which also have a very high false positive rate ^1^. False positive alarms have multiple causes, including ‘low threshold’ settings, motion interference, and false signals generated from a variety of clinical activities. Paradoxically, the high rate of false positive (80–99%) alarms trains practitioners to ignore alarms ^2, 3^. Alarm fatigue is a phenomenon where practitioners come to ignore alarms ^3^. In many ICUs, the audible signals from the alarms built into their bedside monitors are disabled or silenced. This strategy has reduced the noise pollution associated with these systems without obviously decreasing their performance.

Previous literature ^4^ points towards the need to reduce the total number of alarms that occur in working environments such as the ICU. One strategy to increase the clinical utility of such alarms is to specify alarm definitions that are less sensitive, but have a high positive predictive value (PPV). Based on Signal Detection Theory ^5^ strategies to accomplish this could include higher thresholds for alarm conditions, and advanced alarms that might be less likely to be triggered by either artifact or clinical activity. Higher thresholds would alarm less often, but would also alert caregivers later in the course of a patient’s decompensation. Importantly, setting the threshold for an alarm at a higher value may not substantially change the rate of false alarms from artifacts. Alarms with a higher positive predictive value would be triggered less often, and would be much more likely to summon bedside caregivers to respond appropriately. The greatest risk from this strategy is that an alarm might not sound when a life threatening condition is present.

Another strategy to reduce the rate of false alarms is to increase the sophistication of the alarm software ^6^, in effect, making the monitor analyze data across sensors to verify the alarm condition. For example, when a patient moves, she can disturb her EKG electrodes and produce an EKG signal that appears to be ventricular fibrillation. In this instance, the EKG alarms 'V fib'! Frequently, however, other sensors are generating information that could be used to suppress that false alarm.

The correlation of information across sensors may be especially effective in reducing artifact related false alarms. For example, either an arterial line or a pulse oximeter might detect a pulse in the above patient, which is impossible in the setting of V fib. By comparing information across sensors, smarter monitors might decrease the rate of false alarms and facilitate the early detection of other clinical problems. Similarly, a patient who is tachycardic should have a high heart rate on their EKG, pulse-oximeter, and arterial line (if one is present). Simply correlating information from these different sensors is likely to decrease the rate of false alarms without reducing sensitivity to a clinically important degree. The presence of alarms triggered by a single sensor is an artifact of device history, not deliberate design. Advanced software could be programmed to replicate the logic that caregivers utilize to discriminate real conditions from artifact.

Another strategy to increase response to alarms is to assess parameters that are clinically important in the context of the abnormal parameter. For example, tachycardia associated with a precipitous decline in blood pressure is almost always clinically more significant than tachycardia associated with no change or an increase in blood pressure. Advanced alarms which alert bedside caregivers to important patterns of change (clinical correlations) are far more likely to generate the desired clinical response than monitors that continually alarm for situations that represent little or no danger. Such alarms would have a high PPV, lower rate of false alarm, and are likely to elicit more purposeful responses from caregivers.

In this study, we utilized Philip’s Event Monitoring software to define alarm conditions that correlated information across sensors, and which were prospectively intended to have a high positive predictive value. The software being studied in this trial is intended to serve both of these purposes, and the data collected during this trial will inform its refinement.

The Clinical Study of the Event Surveillance Software/Event Alarming usability and functionality is a feedback collection and comparative multi-center study of the recently released Philips' D. O. software for Intellivue Monitors (MP70/90). The software was designed to detect scenarios that are either harmful or might predict a critical situation for the ICU patient.

## Methods

Cardiac surgery patients in a 10 bed Intensive Care Unit were eligible for Intellivue monitor data capture for the purpose of determining the incidence of true positive events as compared with false positive events. IRB approval was obtained and waiver of consent was granted. Event Surveillance software was installed into every monitor in the ICU, and operational in parallel with the institutional default alarms settings. Five clinically important alarm scenarios (‘smart alarms’) were programmed into the bedside monitors using the Event Surveillance software (Table 1).

**Table 1.** Clinical alarm scenarios that were programmed into the bedside monitors.

**Detected Scenarios**		**Parameters**	**Limits/Trigger Time**
***(scenario name)***	*(detect what?)*	*(maximum of four)*	*(lower & upper violation* *for x seconds or relative triggers* *in % over a defined time* *in sec/min)*
**SVT + BP**	onset of paroxysmal atrial fibrillation	HR (Pulse) ART sys Pulse (HR)	+40% within 59 sec -15% within 59 sec >110 bpm for 20 sec
**Vtach + BP**	Vtach with low blood pressure	HR (Pulse) PVC ARTsys Pulse (HR)	+30 bpm within 20 sec ***Vtach -30% within 20 sec >110 bpm for 10 sec
**LV Shock**	left ventricular shock	ARTsys CVPmean PAPdia Perf	<78 mmHg for 300 sec <16 mmHg for 300 sec >16 mmHg for 300 sec <1.2 for 300 sec
**TPX & TPND**	tamponade (obstructive shock)	ARTsys CVPmean Perf PAPdia	<78 mmHg for 180 sec >16 mmHg for 180 sec -20% within 3 min >16 mmHg for 180 sec
**Hypovl**	hypovolemia	ARTmean CVP Perf NIBPm	<50 mmHg for 300 sec <5 mmHg for 300 sec -20% within 120 sec/10 min <55 mmHg for 300 sec

Notes on names in Table 1

1. SVT + BP – Supraventricular Tachycardia and Blood Pressure – This is intended to indicate high heart rate with low blood pressure, as frequently occurs in patients with Atrial fibrillation and a rapid ventricular rate. Tachycardia associated with hypertension, as commonly occurs with light sedation, would not trigger this alarm.

2. VTACH + BP – This is intended to indicate ventricular tachycardia with low blood pressure. This definition would be much less likely to be triggered by motion artifact than the EKG alarm is.

3. LV SHOCK – This is intended to detect Left ventricular failure (cardiogenic shock).

4. TPX & TPND – This is intended to detect either tamponade or tension pneumothorax.

5. HYPOVL – This is intended to indicate low blood pressure from hypovolemia.

The first two (SVT+BP and Vtach+BP) definitions required the presence of an arterial line and EKG. The third and fourth (LV shock and tamponade) required a pulmonary artery catheter and an arterial line. Hypovolemia required the presence of a CVP monitor, and could be triggered by a blood pressure from either the arterial line or a non-invasive blood pressure cuff. If the requisite sensors were not present in a patient, then events and definitions related to that event were not analyzed for the purposes of this study. For example, if atrial fibrillation happened in a patient without an arterial line, it was ignored for the purposes of this study.

When any alarm (factory installed or event surveillance software) is triggered, a log of monitor data from the event is stored in the central monitoring station. Every day, the log file of events from the previous 24 hours was reviewed with the ICU physician (attending or fellow), and all events were classified (Table 2).

**Table 2.** Events’ classifications.

**Abbreviation**	**Explanation**
TPRE	True Positive Real Event
TP Predict	True Positive Predictive
FP Art	False Positive Artifact (e.g. CVP 200 mmHg or Arterial pressure -10 mmHg)
FP Ins Dif	False Positive Insufficient Definition (e.g. patient on LVAD with Vtach or atrial fibrillation)
FN Th	False Negative threat or late (definition failure)
FN No Th	False negative non-threat (e.g. atrial fibrillation without significant hypotension).
FN Sens Off	False Negative sensor off (e.g. atrial fibrillation that occurred while RN was positioning patient and EKG was disconnected).
TN Time Int	Time Interval. These were the patients for which no events were registered during the time period of the observation.

## Results

Events were recorded for 85 days from Mid-May 2007 until Mid-November 2007 (Table 3). In total 564 patient days monitored were monitored.

**Table 3.** Number of true positive, false positive and false negative events, together with the positive predictive value for each clinical alarm scenario using Event Surveillance software.

**Scenario**	**# Events**	**True** **Positives** **(#Patients)**	**False** **Positive** **Artifact**	**False** **Positive** **Insufficient** **definition**	**Positive** **Predictive** **Value**	**False** **Negative**
SVT+BP	221	170(10)	17	22	0.8	9(7)
Vtach+BP	1	1(1)	0	0	1.0	0
LV shock	42	34(6)	8	0	0.81	1
Tamponade	24	1(1)	23	0	0.04	1
Hypovolemia	29	8	21	0	0.27	2

For SVT + BP there were a total of 221 events over 35 patient days. There were 529 patient days where this event did not occur (i.e., no alarm and no false negative occurred).

Out of the 221 events, 170 were True Positive events and 1 was a TP predict event (see Table 2 for abbreviations). 19 were FP Artifact and 22 were FP Insufficient Definition. Thus, out of a total of 221 alarms, 171 were true positive, for a PPV of 0.807.

The 171 TP events were concentrated on 10 patients (patient IDs: 31, 1, 22, 11, 10, 32, 19, 17, 8, 4). The 9 FN events happened to 7 patients. Ventricular Tachycardia with hypotension occurred only in one patient during the 564 recorded patient days, and there were no FP or FN events. Left Ventricular (LV) Shock occurred in 42 of the 564 patient days and among 6 patients in total. There were 8 FP Artifact events and only 1 FN with threat. Thus, the PPV here was 0.81. Tamponade had only one TP event, and 23 FP events (for 13 patient days), as well as 1 Non-threatening FN event in a total of 564 patient days.

The PPV was therefore 0.04. Hypovolemia had 8 TP events, as well as 21 FP events (for 10 patients) and 2 FN events. For Hypovolemia the PPV was 0.27.

## Discussion

No alarm system in use or under development can perform perfectly. Hence, practitioners are compelled to trade-off among the kinds of failures that are acceptable to them. While there is ample literature that demonstrates that simple monitors generate vastly more false alarms than real alarms, the regulatory environment of most medical practice has generated regulations that require these alarms to be activated.

In the current study, the data we have collected thus far suggest that the SVT+BP trigger group is likely to be a useful alarm in clinical practice. The evidence is not quite as strong, but is encouraging for LV shock as well. The other events we were surveying for, tamponade, hypovolemic shock, and Vtach+BP were all sufficiently rare (by our definition) that we remain unable to evaluate the positive predictive performance of these trigger groups. While LV shock is commonplace in the ICU where this study was conducted, most patients were actively managed by their caregivers and rarely met the definition for LV shock we employed. Importantly, the absolute rate of false positive alarms for these groups was low (29%) compared to the approximately 80% rate reported in other studies ^2^, consistent with our hypothesis that correlating information across sensors might decrease the rate of false positive alarms. Correlating information across sensors and simultaneously probing for important deflections from other sensors produced a dramatic improvement in alarm performance in this study.

The most important limitation to this approach is that event surveillance software utilizing multiple sensors requires that those sensors be present, operational, and free of artifact. There were multiple episodes of atrial fibrillation that occurred in patients who did not have an arterial line, and were hence not captured by event surveillance software, and not eligible for inclusion in this analysis. Dampening of the arterial waveform produced a situation in which the criterion for hypotension was satisfied in event surveillance software. This was principally a problem with the SVT+BP and hypovolemia definitions, but would confound any definition that relies upon accurate data from an arterial catheter. Another important failure came from artifact in the CVP. Failure to level can produce artifactually high or low values in the CVP. Infusions consistently produce artifactually elevated CVP measurements. These artifacts generated most of the false positives in the hypovolemia and tamponade definitions. The software used to conduct this study did not allow any parameter from a sensor to be used more than once in any definition, which precluded screening for these artifacts by excluding extreme values (e.g. CVP of 60mm Hg or -20 mmHg). The ability to examine a parameter more than once would have prevented many of the false positive activations of these definitions. The failure rate of definitions that require data from different sensors will be at least the sum of the artifact rate of those sensors. Logic that replicates how human operators process alarms can be employed using Event Surveillance software and similar software, and has the potential to significantly improve the performance of bedside monitors.

The event surveillance software employed in the present study could not access all of the information generated from all of the sensors in the monitor, which severely constrained the events that could be surveyed and the definitions that were generated. Successive generations of software, if they incorporate expanded ability to capture information, might be used to generate definitions that will be more useful than most of those used for the current study.

The most important limitation of the present study is that we were unable to deploy an independent observer in the ICU continuously, and thus had to depend upon bedside RNs and resident physicians to report episodes of the events we sought to capture. It is unlikely that we missed a large number of significant events, but precise estimation of the performance of these definitions would require this more reliable database. We hope that we will be able to obtain the resources to perform a successor study of this design at multiple sites. If all of the output from the clinical devices was recorded into a single massive database, that database could then be used to iteratively evaluate and refine different alarm definitions.

Event surveillance software utilizes the same audible and visible signals as the other alarms built into these monitors. Hence, study definitions with a very high true positive alarm rate were mixed in with the high rate of false alarms generated by the factory settings for each sensor. The number of false alarms from the individual sensors substantially outnumbers the alarms generated by event surveillance software. Until such time as different audible and visual alarms are utilized, it may be difficult or impossible to demonstrate an important difference in the response of bedside caregivers.

## Conclusion

Correlation of information across sensors can be used to detect and suppress artifact in a manner similar to how human operators analyze data. Such simple algorithms can generate alarms with a much higher positive predictive value than the simple alarms associated with any of the individual sensors. Additionally, the ability to correlate information across sensors allows the monitor to process clinical information in a manner similar to human operators. The most important limitation to the correlation of information across sensors is that the failure rate becomes at least the sum of the artifact rate of the individual sensors. Nevertheless, these two approaches have the potential to significantly reduce false alarms, increase the positive predictive value of alarms, and make some progress reducing the ubiquitous problem of alarm fatigue in the ICU.
