# Supplementary material for: Correlating data from different sensors to increase the positive predictive value of alarms: an empiric assessment
Source: F1000Res. 2012 Nov 8;1:45. [Version 1] doi: 10.12688/f1000research.1-45.v1 (PMC3752621; doi:10.12688/f1000research.1-45.v1)
Supplement: Peer review history

# Peer review: Referee response for version 1

**Published:** 2012-11-27

**Authors:** Gorazd Voga

**Affiliations:** Medical ICU, General Hospital Celje, Celje, Slovenia

The ideology behind the research of this article is good and relevant. Despite the article having a few flaws, the work presented highlights an important topic that is worthy of further discussion.

I have read this submission. I believe that I have an appropriate level of expertise to confirm that it is of an acceptable scientific standard.

# Peer review: Referee response for version 1

**Published:** 2012-11-19

**Authors:** Melanie Wright

**Affiliations:** Trinity Health System, Boise, ID, USA

The scope and depth of the work is appropriate as something that would be presented as an abstract or pilot work, as the study is a collection of baseline data.

There are no comparisons of other methods used to monitor patients, for example, did the authors turn off the single sensor alarms whilst performing this study? The authors also compare their presumed false alarm rates to rates presented in other studies, rather than actually capturing single sensor false alarm rates in this setting, and it is difficult to understand how one might place the use of the correlating data (for example SVT + BP to detect atrial fibrillation) within the context of other conditions that low BP and/or high HR/pulse might predict. How did they determine false negatives? Expert review of alarm logs does not instill me with confidence that they captured events that may have been missed. I think the limitations, appropriately described within the document, are great enough to question whether this research is yet at a level that is meaningful for a wide audience. However, the writing is good and the findings may be meaningful for others working in this developing area of research.

I have read this submission. I believe that I have an appropriate level of expertise to confirm that it is of an acceptable scientific standard, however I have significant reservations, as outlined above.

# Peer review: Referee response for version 1

**Published:** 2012-11-15

**Authors:** Yan Xiao

**Affiliations:** Office of Patient Safety, Baylor University Medical Center at Dallas, Dallas, TX, USA

I have read this submission. I believe that I have an appropriate level of expertise to confirm that it is of an acceptable scientific standard.
